# Water stress effect on hydraulic architecture, biomass partitioning, and gas exchange of four different olive cultivars

**DOI:** 10.3389/fpls.2025.1630454

**Published:** 2025-08-18

**Authors:** Valeria Imperiale, Tiziano Caruso, Antonino Ioppolo, Alessandro Carella, Roberto Massenti, Francesco Paolo Marra

**Affiliations:** Department of Agricultural, Food and Forest Sciences, University of Palermo, Palermo, Italy

**Keywords:** *Olea europaea* L., HPFM, transpiration, climate change, drought stress, stem water potential, tree physiology

## Abstract

*Olea europaea* L. is considered a very resilient species to water deficits. Climate change, characterized by warmer summers and drier winters, may challenge even this adaptable species, potentially making once-suitable areas less viable for cultivation. Identifying cultivars with enhanced drought tolerance is essential for the future of olive growing. This study aimed to evaluate the water stress response of four olive cultivars: Biancolilla, Calatina, Nocellara del Belice, and Koroneiki, by analyzing their physiological traits and hydraulic properties. Potted plants were subjected to three irrigation treatments: T20, T50, and T100, corresponding to 20%, 50%, and 100% of crop evapotranspiration over approximately two months. Midday stem water potential and gas exchange were monitored throughout the trial. At the end of the experiment, plants were analyzed using a High-Pressure Flowmeter (HPFM) to measure hydraulic conductance (k) in leaves, shoots, trunks, and roots, providing insights into the plant’s hydraulic architecture. On these bases, the study aimed to identify strategies that different cultivars use to handle water stress. Biomass distribution and growth were significantly affected by cultivar and irrigation, with reductions occurring under severe stress, particularly in the shoots and leaves, while root biomass remained relatively stable. The relative stability of the root system and changes in canopy-to-root ratios highlight adaptive responses aimed at maintaining water uptake and ensuring stress resilience. ‘Calatina’ and ‘Nocellara del Belice’ showed less sensitivity to irrigation levels, maintaining relatively stable dry matter across all organs and treatments, while ‘Biancolilla’ and ‘Koroneiki’ exhibited the opposite. Different relative allocation strategies were evident: ‘Calatina’ prioritized shoot and leaves biomass and showed dwarfing growth; ‘Biancolilla’ invested more in trunk development, as well as ‘Nocellara del Belice’; ‘Koroneiki’ focused more resources on roots. Distinct hydraulic strategies emerged among the cultivars: ‘Koroneiki’ maintained high conductance in aerial parts, supporting sustained photosynthesis and growth; ‘Calatina’ adopted a conservative approach, prioritizing root conductance and limiting transpiration; while ‘Biancolilla’ and ‘Nocellara del Belice’ exhibited intermediate, balanced responses. These findings confirm the strong link between hydraulic architecture and physiological performance, offering insight into cultivar-specific responses to water stress and growth potential.

## 1 Introduction

Olive (*Olea europaea* L.) is widely renowned for its remarkable resilience and ability to tolerate various abiotic stresses, which makes it a highly valuable crop, especially in the Mediterranean region. This area is characterized by severe environmental conditions such as water scarcity, extreme temperatures, and intense solar radiation, all of which pose significant challenges to agricultural practices. Olive trees, however, have evolved numerous adaptive mechanisms to thrive under such conditions (Fernández, 2014; Pérez-Pastor et al., 2016; Marino et al., 2021; Massenti et al., 2024). These unique characteristics have made the olive tree a source of Mediterranean agriculture for thousands of years, contributing not only to the local economy but also to the cultural and historical identity of the region (Besnard et al., 2018).

Despite the natural adaptability of the olive tree, the increasing impact of climate change in recent years has raised concerns, particularly regarding the sustainability of olive cultivation (Fraga et al., 2020). Climate models predict rising temperatures, reduced rainfall, and prolonged drought periods in the Mediterranean basin, all of which will exacerbate the challenges faced by olive farmers. In southern Italy, for instance, minimum temperatures have increased, while rainfall has decreased, resulting in water shortages that have become increasingly problematic. In Sicily, emergency measures were declared due to water scarcity as early as February in consecutive years, until 2024. According to the Copernicus Climate Change Service (C3S) (Copernicus Climate Change Service, 2024), global temperatures in 2024 approached the critical threshold of +1.5 °C, making it the hottest year on record. Furthermore, this year saw several extreme weather events, including heatwaves and wildfires, all of which contribute to an unstable environment for olive cultivation. Under current climate change projections, prolonged drought events are expected to become more frequent and intense, leading to significant losses of crop yields and quality, as well as changes in the geographic distribution of olive orchards (Haokip et al., 2020; Rodrigo-Comino et al., 2021; Imperiale et al., 2022).

Considering these challenges, it has become increasingly important to understand the physiological mechanisms by which olive trees cope with environmental stress. Olive trees are known for their exceptional longevity and stress tolerance, which allows them to endure extreme conditions that would be lethal to many other crops. Olive trees can maintain satisfactory yields and high-quality olive oil production until reaching water potential as low as -3.5 MPa (Marra et al., 2016; Ahumada-Orellana et al., 2019; Marino et al., 2018; Massenti et al., 2024), and survive even more severe water stress down to -6.0 MPa (Baccari et al., 2020), thanks to their highly efficient stomatal regulation and water strategy (Dichio et al., 2006; Fernández, 2014).

A key factor in the olive hydraulic strategy under drought stress is the ability to maintain xylem water transport efficiency. Some olive cultivars exhibit narrower and more densely packed xylem vessels, which confer greater resistance to cavitation and hydraulic dysfunction (Torres-Ruiz et al., 2013; Sebastiani et al., 2016). This adaptation allows certain olive cultivars to sustain hydraulic conductivity even under severe drought conditions, enhancing their overall resilience.

Beyond their physiological adaptations, the species also exhibit morphological traits that help them cope with environmental stress, particularly drought. Olive leaves are highly adaptable and can undergo changes in the thickness, shape, and density of trichomes and stomata to minimize water loss through transpiration, thereby enhancing the water retention capacity of the plant (Bosabalidis and Kofidis, 2002; Khalil and El-Ansary, 2020). These morphological adjustments support the tree's overall resilience in extreme weather conditions.

Advancements in the field have led to the establishment of new super-high-density (SHD) olive growing systems, which aim to improve productivity and reduce labor costs, such as those implemented in Spain and other new olive-growing regions like the USA, Argentina, and Australia (Serman et al., 2021; Fernández et al., 2022; Sobreiro et al., 2023; Caruso et al., 2023). These systems involve the use of low-vigor, compact cultivars, which are better suited to mechanized farming practices.

Although numerous studies have examined regulated deficit irrigation (RDI) strategies in traditional olive orchards (Moriana et al., 2003; Iniesta et al., 2009; Ramos and Santos, 2009), their findings may not be directly applicable to super high-density (SHD) systems. In SHD orchards, trees often develop smaller root systems (Díaz-Espejo et al., 2012), which can increase their vulnerability to water stress compared to trees in conventional plantings. The combination of dense canopies, resulting in greater water demand and the limited soil volume available per tree means that irrigation is typically essential in SHD systems (Rosecrance et al., 2015; Arbizu-Milagro et al., 2022; Sobreiro et al., 2023).

This raises concerns about their long-term sustainability in the face of increasing water scarcity and climate variability. In traditional countries like Italy, where olive oil production is rooted in traditional methods and local cultivars, such systems may not be immediately applicable (Marino et al., 2019; Lo Bianco et al., 2021). Consequently, research on controlling tree vigor using rootstocks and other agronomic techniques has gained importance, with the goal of improving olive cultivation practices without compromising the distinctive qualities of traditional olive oils (Rodrigues et al., 2018; Camposeo et al., 2021; Grilo et al., 2021; Massenti et al., 2022). Additionally, there is a growing emphasis on selecting cultivars that can endure extreme environmental conditions, such as prolonged droughts and high temperatures which are becoming more frequent due to climate change, while also reducing irrigation inputs (Massenti et al., 2022; Granata et al., 2025). This study aims to evaluate the eco-physiological and morphological response of four different olive cultivars to prolonged water stress, to deeper understanding how different cultivars respond to the increasing abiotic stresses associated with climate change. It will also provide insights into the development of more resilient olive cultivars, better equipped to handle the challenges posed by global warming, supporting the long-term sustainability of olive farming in the Mediterranean region.

## 2 Materials and methods

### 2.1 Plant material and experimental design

The experiment was conducted from September to late November 2022 in a greenhouse at the Agricultural, Food, and Forest Sciences (SAAF) Department of the University of Palermo (38°06’24”N, 13°21’01”E). The greenhouse conditions were designed to accelerate transpiration, induce water stress in the plants, and subsequently evaluate their drought resistance. Two-year-old potted trees from three local olive cultivars, Biancolilla, Calatina, and Nocellara del Belice (which, for simplicity, will hereafter be referred to as ‘Nocellara’) and one international, Koroneiki, were studied. 15L pots were used with 50% sandy loam soil and 50% perlite, to allow the easy removal of the loam from the roots without breaking them. Each pot was covered with a plastic bag to prevent soil evaporation and isolate transpiration as the only cause of water loss. Three different irrigation treatments—T100, T50, and T20—were applied to simulate the effects of drought stress. In detail, 100, 50, and 20% of the transpired water were added, respectively.

Before starting the trial, each pot was saturated with water and allowed to drain for 24 hours, after which its weight was recorded as field capacity. For each experimental unit, the maximum amount of water retained at field capacity was calculated by subtracting the pot weight at transplanting. Based on the field capacity, weight thresholds were established for T50 and T20 treatments, calculating 50 and 20% of the weight at field capacity, respectively. The well-watered plants (T100) were weighed daily and rehydrated to field capacity. In contrast, stressed ones (T50 and T20) were weighed every two days and rehydrated when their weight fell below the pre-established threshold. The pots were arranged in a randomized block design, with drought stress levels (three) and cultivars (four) as main factors. Six replicates per treatment were used, eighteen plants per cultivar. Moreover, biometric measurements were taken, including plant height and trunk diameter (30 cm above the root collar), and the number of leaves was counted.

### 2.2 Transpiration, gas exchanges, and water potential

During the trial, a datalogger (WatchDog) was installed in the greenhouse to monitor temperature and relative humidity, enabling the calculation of vapor pressure deficit (VPD). Transpiration was monitored on all the plants in the study (eighteen per cultivar) by weighing the pots to maintain them at the established threshold. Hence, daily transpiration was standardized by the canopy surface area of each plant (SDT; g_H_2_O_ d^-1^ m^-2^) to evaluate plant responses to water stress.

Stomatal conductance (gs; mmol H₂O m⁻² s⁻¹), net assimilation (An; μmol CO₂ m⁻² s⁻¹), substomatal CO₂ concentration (Ci; μmol mol⁻¹), and intrinsic water use efficiency (WUE_i_; μmol CO₂ mmol⁻¹ H₂O) were measured every two weeks on fully active and expanded leaves on three plants per treatment and cultivar (N=36). The mesophyll efficiency (C_i_/g_s_) was also calculated to compare treatments and cultivars according to Sheshshayee et al. (1996). Three leaves per experimental unit were analyzed using a gas exchange analyzer (CIRAS-3, PPSystem, Hitchin, UK). Measurements were taken at greenhouse temperature, using natural light on sunny days. The LED light unit was used on cloudy days, set over the saturation threshold of 800 µmol m^−2^ s^−1^, CO_2_ concentration was set at 400 µmol mol^-^¹, the air flow rate at 200 ml min^-^¹, and the relative humidity was maintained at 80% of the ambient level. Midday stem water potential (Ψ_stem_) was measured on the same plant as the gas exchange measurements. One leaf per experimental unit was covered with aluminum foil to prevent transpiration, allowing it to equilibrate with the stem water potential for at least 1 hour before measurements, which were taken at solar noon (between 12:00 and 15:00 local time) using a Schölander pressure chamber (PMS 1000, Instrument Company, Albany, OR, USA; Scholander et al., 1965). Finally, to account for the fluctuations in the plants’ water status, cumulative Ψ_stem_ was calculated by numerical integration using the trapezoidal method proposed by Gucci et al. (2007) with the equation:


$${\text{Cumulative Ψ}}_{\text{stem}}\;=\sum_{\text{DOY }334}^{\text{DOY }261\;}\frac{\left({\text{Ψ}}_{{\text{stem}}_{1}}+{\text{ Ψ}}_{{\text{stem}}_{2}}\right)\text{x}\left({\text{t}}_{2}-{\text{t}}_{1}\right)}{2},\;\left(\mathrm{MPa*d}\right)$$

where Ψ_stem1_ and Ψ_stem2_ are Ψ_stem_ values (MPa) on two consecutive dates, and (t_2_ – t_1_) the time interval expressed in days (DOY).

### 2.3 Hydraulic conductance

At the end of the drought stress trial, hydraulic conductance (k) of the roots, trunk, shoots, and leaves was measured on the same plants as the gas exchange measurements and Ψ_stem_, using a High-Pressure Flow Meter (HPFM – Dynamax, Houston, Tex.) as described by Tyree et al. (1994, 1995).

Ψ_stem_ was recorded the day before hydraulic conductance measurements, and any wounds caused by leaf excision were sealed with glue. To minimize the potential effects of diurnal periodicity on plant hydraulic conductance (Henzler et al., 1999; Tsuda and Tyree, 2000; Nardini et al., 2006), all measurements were conducted in a laboratory under controlled environmental conditions using LED lighting from a growth chamber. The first measurement was performed on the root system. The aerial part of the plant was excised 5 cm above the root collar, and the cut surface was immersed in water. The leaves were sprayed with water, and the entire canopy was enclosed in a transparent plastic bag to minimize transpiration (Nardini et al., 2006; Bogeat-Triboulot et al., 2002). The root collar was then recut under distilled water to prevent xylem embolism and connected to the HPFM collector (Nardini and Tyree, 1999).

Given the high regenerative capacity of the root system, transient measurements were preferred over the quasi-steady state mode to enable rapid data acquisition and minimize errors (Nardini et al., 2006; Raimondo et al., 2005). In the quasi-steady state mode (constant flow at constant pressure), water flow may decrease as it reaches the root tips, potentially compromising measurement accuracy. This approach minimizes potential errors, by perfusing water in the opposite direction of root absorption (Tyree et al., 1994 and 1995).

For each plant, three to five measurements of k_root_ were taken. Water flow (F) was measured at increasing pressure (P) ranging from 0 to 500 kPa at a constant rate of 0.5 kPa s^-1^, while degassed distilled water (DDW) was perfused through the root system. k was calculated as the slope of the plot of F *versus* P,


$$\text{k}=\text{ΔF}/\text{ΔP},\;\left({\text{kg s}}^{-1}{\text{ MPa}}^{-1}\right)$$

The HPFM was then connected to the base of the bagged stem and DDW was perfused at a constant pressure of 500 kPa using the quasi-steady state mode measurement until the flow rate stabilized (approximately 2 hours). Subsequently, canopy conductance (k_canopy_) was determined using three transient measurements (Yang and Tyree, 1994). Next, the leaves were removed, and three additional transient measurements were performed to determine the conductance of the leafless stem (k_residual_). Leaf hydraulic conductance (k_leaf_) was then calculated as:


$${\text{k}}_{\text{leaf}}={\text{k}}_{\text{canopy}}-{\text{k}}_{\text{residual}}$$

Following this, the shoots of the main trunk were excised and the k_trunk_ was measured. The shoots (k_shoot_) hydraulic conductance was calculated as follows:


$${\text{k}}_{\text{shoot}}={\text{k}}_{\text{canopy}}-{\text{k}}_{\text{leaf}}-{\text{k}}_{\text{trunk}}\;$$

All hydraulic conductance measurements were normalized by the corresponding biometric parameters: leaf area of the tree for the k_leaf_, length for the k_shoot_ and k_trunk_, and dry matter for the k_root_.

### 2.4 Biomass partitioning

At the end of the trial, after hydraulic conductance measurements, the fresh weight of each part of the plant (roots, trunk, shoots, and leaves) was recorded. The roots were removed from the pots and carefully washed, with special care taken to recover all roots using sieves of different mesh sizes. The leaves were counted, along with the lengths of the trunks and shoots, and the diameter of the trunk was recorded. Finally, all plant parts were oven-dried at 80°C until a constant weight was achieved. Before drying, a sample of 30 leaves per plant was scanned to determine the single leaf area using ImageJ/Fiji software (version 2.14.0).

The canopy leaf area of each plant was measured by multiplying the number of leaves by the average area of the single leaves. Additionally, the canopy-to-root mass ratio and the relative biomass allocation for the leaves, shoots, trunks and roots were calculated.

### 2.5 Statistical analysis

All statistical analyses were performed using the general linear model to assess the effects of cultivar, irrigation treatment, and their interaction on physiological, biometric, and hydraulic parameters. When significant differences were detected (P ≤ 0.05), means were separated using Tukey’s HSD *post hoc* test. Linear and non-linear regression analysis were also performed. All statistical analyses were conducted using SYSTAT (Systat Software Inc., San Jose, CA, USA).

## 3 Results

### 3.1 Environmental conditions

**Figure 1:**
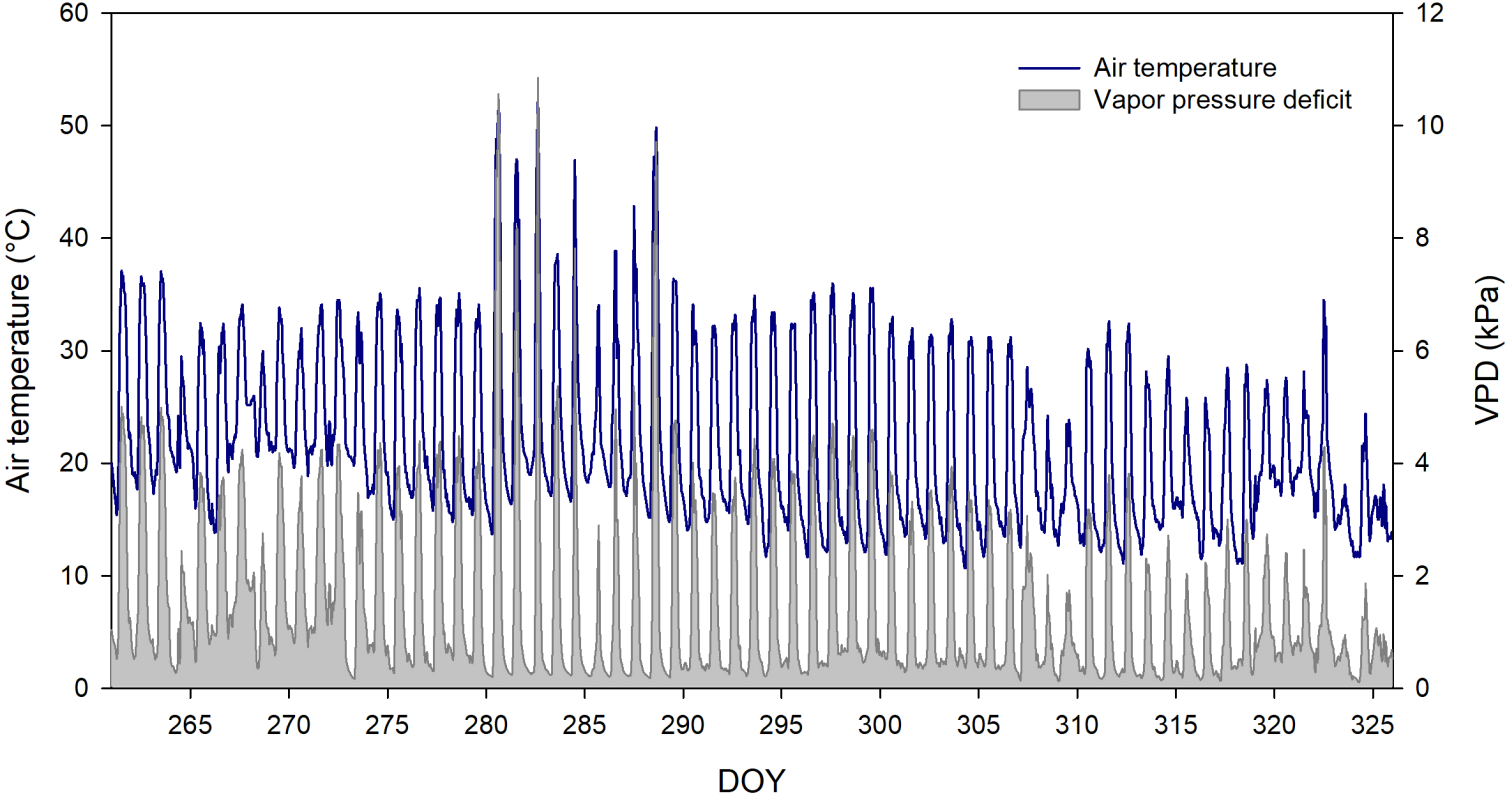
Daily trend of air temperature and vapor pressure deficit during the water stress trial.

The trial lasted 65 days, during which plants were exposed to consistently high ambient temperatures (**Figure 1**). Notably, heatwaves occurred from October 8th to 16th (days 280-288), with air temperatures peaking at 50°C and a VPD reaching 11 kPa. For most of the experiment, environmental conditions remained stable, with maximum air temperatures ranging between 34°C and 37°C until early November. After that, temperatures gradually declined but remained relatively high, fluctuating between 28°C and 31°C.

### 3.2 Effect of water stress on Midday Stem Water potential

**Figure 2:**
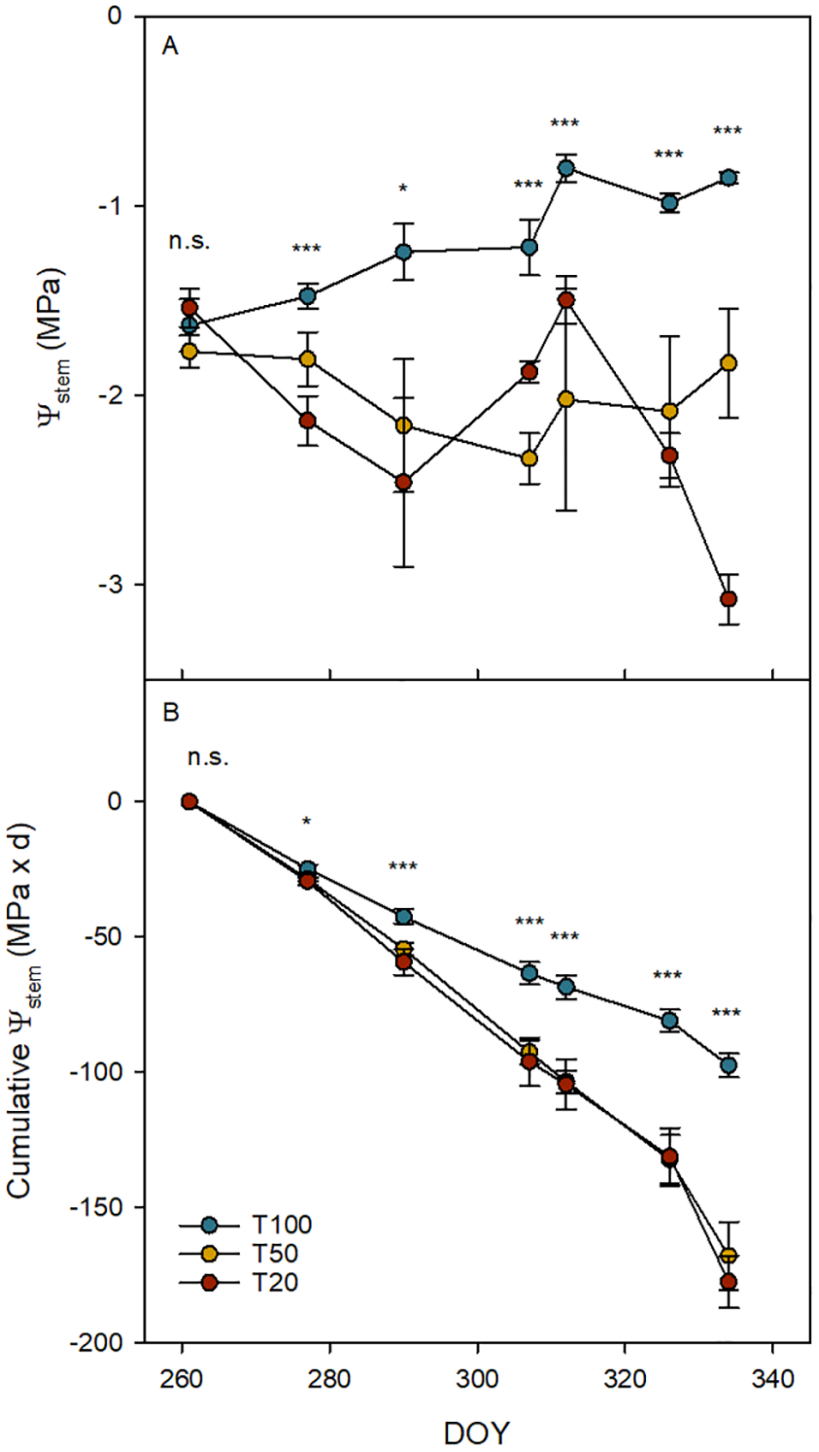
Trends of midday stem water potential (Ψ_stem_, **A)** and cumulative Ψ_stem_ **(B)** on average per treatment. Blue, yellow, and red dots represent the treatments T100, T50, and T20, respectively. Error bars indicate standard errors of means. n.s., not significantly different; *, significantly different for P ≤ 0.05; ***, significantly different for P ≤ 0.001.

Throughout the observation period (DOY 261–334), the different water regimes used in this experiment had a significant effect on the midday Ψ_stem_ for all cultivars, with notable differences between the well-irrigated treatment (T100) and the stressed treatments (T50 and T20). At the beginning of the trial, all plants were fully irrigated to field capacity, and no significant differences were observed among the treatments on DOY 261 (**Figure 2A**). Then, statistically significant differences were found between the treatments (DOY 277); however, in general, both T50 and T20 cumulated a similar amount of stress (**Figure 2B**). The last date indicated represents the Ψ_stem_ at which the hydraulic measurements were conducted. No significant differences in midday Ψ_stem_ and cumulative Ψ_stem_ were observed among cultivars.

### 3.3 Effect of water stress on the biomass partitioning and morphological traits

**Figure 3:**
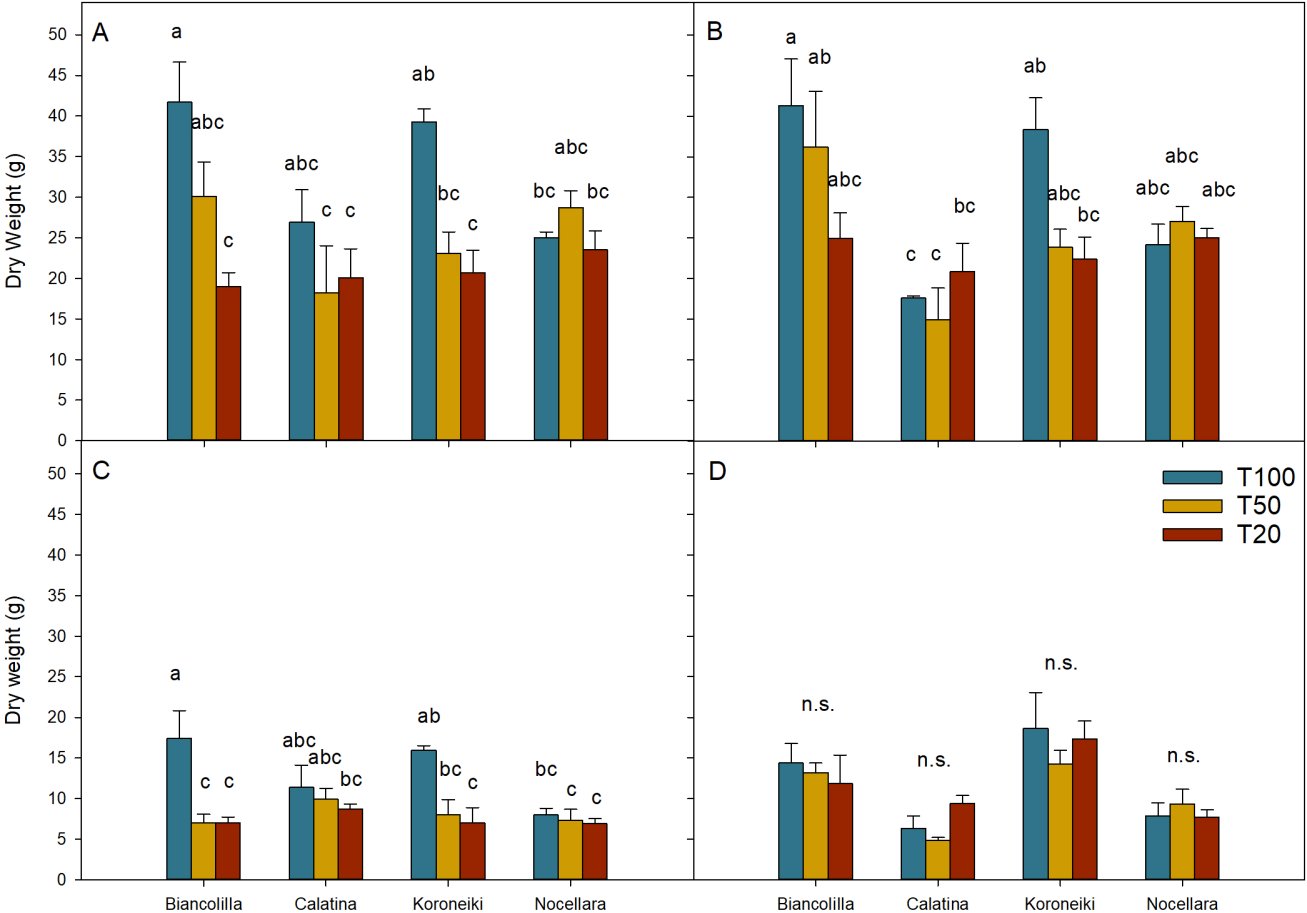
Dry weight partitioning of the leaves **(A)**, trunk **(B)**, shoots **(C)**, and roots **(D)**. Blue, yellow, and red bars represent the treatments T100, T50, and T20, respectively. Error bars indicate standard errors of means. n.s., not significantly different; letters, significantly different in the interaction between cultivars and treatments for P ≤ 0.05.

Biomass distribution and plant growth were significantly affected by the interaction between cultivar and irrigation treatment, with distinct patterns observed across the leaves, shoots, and trunk (**Figure 3**). Under full irrigation (T100), both ‘Biancolilla’ and ‘Koroeniki’ showed the highest total dry biomass, with most of the dry matter allocated to the leaves, followed by the trunk, shoots, and roots. However, in these cultivars, as water availability decreased (T50 and T20), a progressive reduction in dry matter was observed in the leaves and trunks, reaching reductions of almost 50% and 40%, respectively, under T20 (**Figures 3A, B**). Shoots (**Figure 3C**) were even more sensitive, showing almost 60% reduction in both stressed treatments compared to T100. Unlike ‘Biancolilla’ and ‘Koroneiki’, the biomass of ‘Calatina’ and ‘Nocellara’ appeared less sensitive to irrigation levels, maintaining relatively stable dry matter across all organs and treatments, including T100. Although the interaction between the cultivar and irrigation treatments did not result in statistically significant differences in roots (**Figure 3D**), statistically significant differences were observed among the cultivars (P ≤ 0.001). ‘Koroneiki’ had the most developed root system with 16.76 ± 1.64g, while ‘Calatina’ had the least, 7.10 ± 0.93g; cultivars Biancolilla and Nocellara reported intermediate values with 13.15 ± 1.33g and 8.33 ± 0.79g, respectively (**Figure 3D**). The relative allocation of biomass among plant organs varied depending on both cultivar and irrigation treatment (**Table 1**). Under optimal water supply conditions (T100), it was observed that olive plants tend to allocate more resources to leaf production. In contrast, under stress conditions (T50 and T20), dry matter was increasingly allocated to the root system. Regarding cultivar-specific traits, ‘Calatina’ and ‘Nocellara’ allocated more biomass to the canopy. However, ‘Calatina’ invested less in the trunk and more in the shoots, conversely, ‘Nocellara’ and ‘Biancolilla’ concentrated their biomass primarily in the trunk. Unlike all the cultivars, ‘Koroneiki’ invested in root biomass.

**Table 1:** Mean values (± standard error) of relative partitioning of dry weight in the principal organs of the trees at the end of the trial.

Plant organs		Leaves (%)				Shoots (%)				Trunk (%)				Roots (%)			
		Means		SE		Means		SE		Means		SE		Means		SE	
Cultivar	Biancolilla	33.86	±	1.27	ab	11.68	±	1.57	b	38.93	±	1.49	a	15.53	±	1.58	ab
	Calatina	37.99	±	2.09	ab	17.56	±	1.34	a	31.71	±	1.67	c	12.74	±	1.74	b
	Koroneiki	33.07	±	1.14	b	11.94	±	1.07	b	33.99	±	0.72	bc	20.99	±	1.78	a
	Nocellara	38.48	±	1.06	a	11.04	±	0.55	b	37.97	±	0.77	ab	12.50	±	1.16	b
Treatment	T100	38.31	±	1.34	a	14.95	±	1.13	n.s.	33.97	±	1.40	n.s.	12.77	±	1.17	b
	T50	36.08	±	1.22	ab	11.85	±	1.60	n.s.	36.43	±	1.30	n.s.	15.64	±	1.68	ab
	T20	33.00	±	1.13	b	11.89	±	0.80	n.s.	36.95	±	1.17	n.s.	18.16	±	1.79	a
Cultivar*Treatment		n.s.				n.s.				n.s.				n.s.			

Different letters indicate statistically significant differences between means according to Tukey’s HSD test (P ≤ 0.05); n.s., not significantly different.

**Figure 4:**
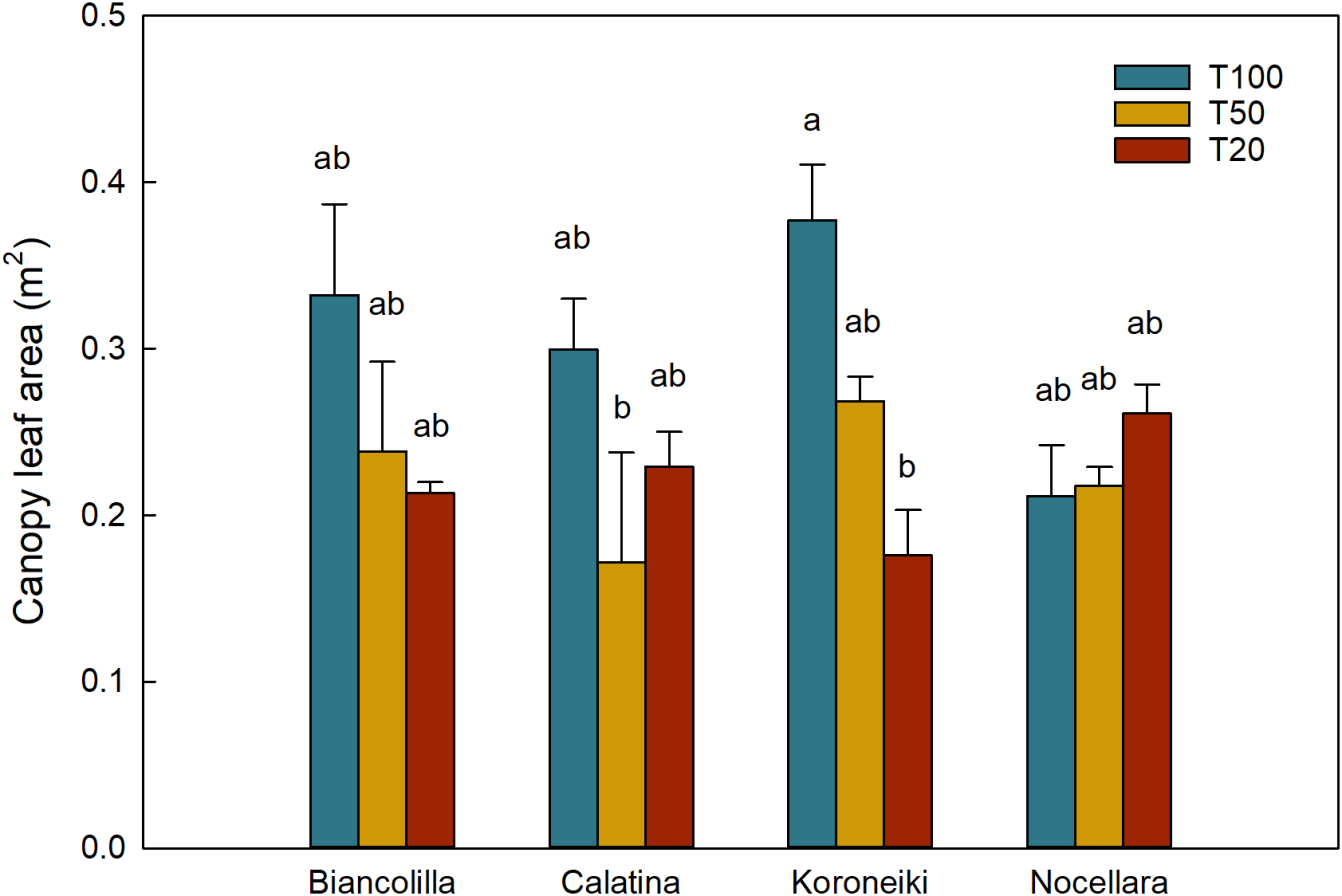
Canopy leaf area (m_2_) of the interaction between cultivar and irrigation treatment. Blue, yellow, and red bars represent the treatments T100, T50, and T20, respectively. Error bars indicate standard errors of means. Different letters mean significant differences for P ≤ 0.05.

Leaf number and leaf surface area were significantly influenced by both cultivar and irrigation level (**Table 2**). In detail, ‘Biancolilla’ produced the highest number of leaves but with the smallest average surface, while ‘Calatina’ and ‘Nocellara’ showed the opposite trend, producing fewer but larger leaves. This compromise resulted in a statistically similar total leaf area among cultivars, indicating a balance between number and size. Water availability further shaped these traits: well-irrigated plants developed more and larger leaves, while stressed plants (T50 and T20) showed clear reductions. The interaction between cultivar and irrigation treatment significantly affected leaf surface area, particularly in ‘Koroneiki’ (**Figure 4**).

**Table 2:** Mean values (± standard error) of morphological parameters at the end of the trial.

Parameters		Leaf number				Leaf area (cm^2^)				Shoots lenght (m)				Canopy/Roots (DW; g)			
		Means		SE		Means		SE		Means		SE		Means		SE	
Cultivars	Biancolilla	743.6	±	82.2	a	3.52	±	0.05	c	7.04	±	0.99	ab	2.45	±	0.36	ab
	Calatina	504.8	±	44.8	b	4.71	±	0.22	a	5.74	±	0.62	ab	3.57	±	0.64	a
	Koroneiki	666.7	±	72.3	ab	4.07	±	0.07	b	7.34	±	0.92	a	1.74	±	0.24	b
	Nocellara	476.6	±	32.6	b	4.86	±	0.12	a	5.12	±	0.38	b	3.3	±	0.31	a
Treatment	T100	716.4	±	76.8	a	4.43	±	0.19	a	8.38	±	0.78	a	3.39	±	0.41	a
	T50	569.4	±	55.7	b	4.07	±	0.18	b	5.74	±	0.42	b	2.71	±	0.41	ab
	T20	513.3	±	28.2	b	4.32	±	0.2	ab	4.81	±	0.30	b	2.11	±	0.29	b
Cultivar*Treatment		n.s.				n.s.				n.s.				n.s.			

Different letters indicate statistically significant differences between means according to Tukey’s HSD test (P ≤ 0.05); n.s., not significantly different.

**Figure 5:**
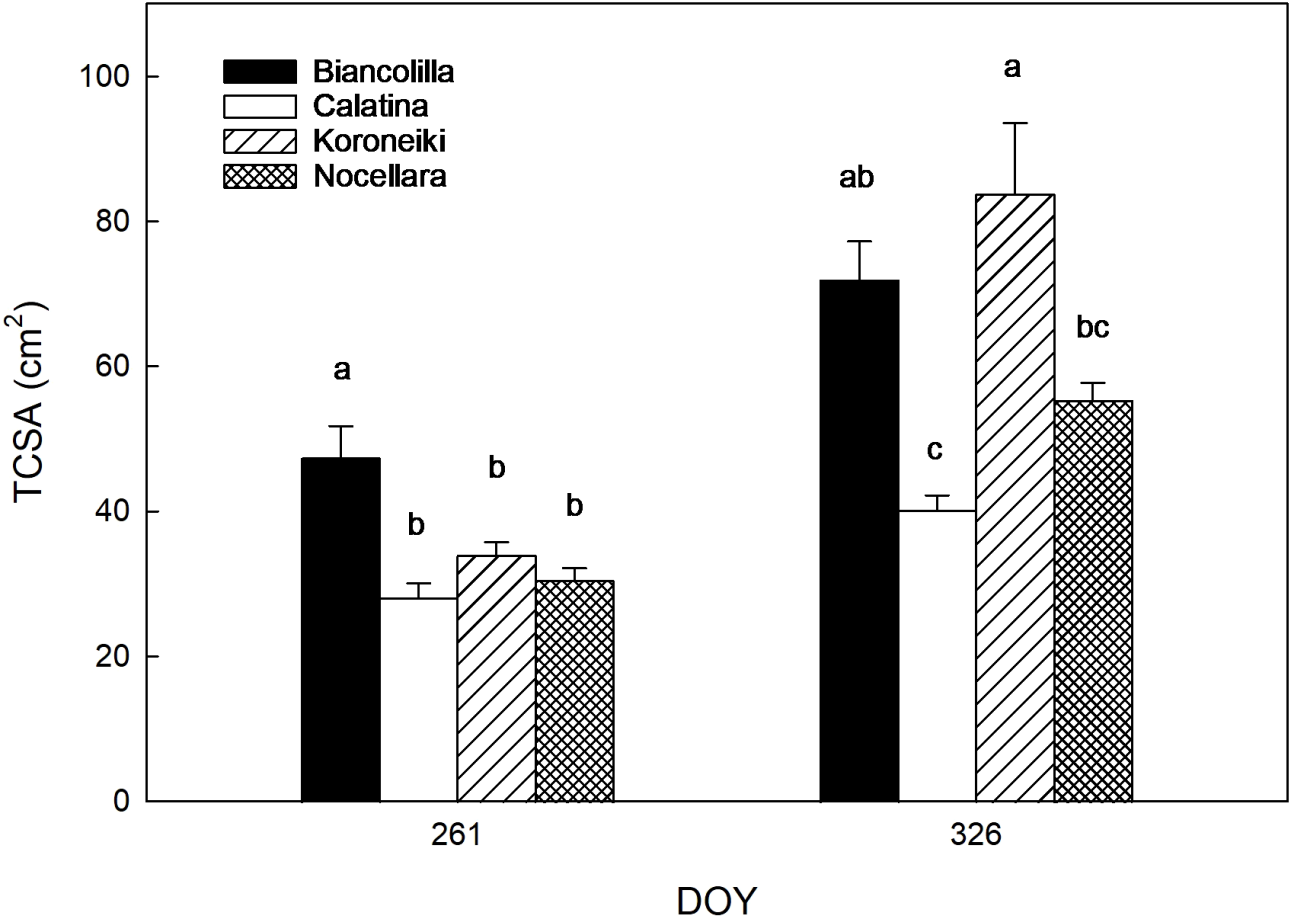
Trunk cross-section area (TCSA) per cultivar at the beginning (DOY 261) and at the end (DOY 326) of the trial. Error bars indicate standard errors of means. Different letters mean significant differences for P ≤ 0.05.

Differences in trunk cross-sectional area (TCSA) also confirmed cultivar vigor levels (**Figure 5**). ‘Calatina’, showed minimal TCSA change between the start (DOY 261) and end (DOY 326) of the experiment. ‘Biancolilla’, confirmed its status as the most vigorous cultivar among the autochthonous varieties, followed by ‘Nocellara’. ‘Koroneiki’ exhibited the largest increase by the end of the trial, surpassing even ‘Biancolilla’.

Statistically significant differences were observed among the cultivars in total shoot length (P ≤ 0.01; **Table 2**). ‘Koroneiki’ exhibited the longest shoots (7.34 ± 0.92 m) while ‘Nocellara’ (5.12 ± 0.38 m) the shortest ones, and ‘Biancolilla’ and ‘Calatina’ showed intermediate values (7.04 ± 0.99 m and 5.74 ± 0.62 m, respectively). Irrigation treatment also influenced shoot growth, with longer shoots, on average, observed in the T100 treatment (8.38 ± 0.78 m) compared to the water-stressed treatments (5.74 ± 0.42 m for T50 and 4.81 ± 0.30 m for T20; P ≤ 0.001.

Water scarcity significantly influenced the canopy-to-root mass ratio, as shown in **Table 2**. The well-irrigated plants (T100) had the highest ratio, which progressively decreased under reduced irrigation (T50 and T20). Statistically significant differences were also observed among cultivars: Calatina and Nocellara showed lower ratios than Koroneiki, while Biancolilla did not differ significantly from either group.

### 3.4 Effect of water stress on gas exchanges and transpiration rate

**Figure 6:**
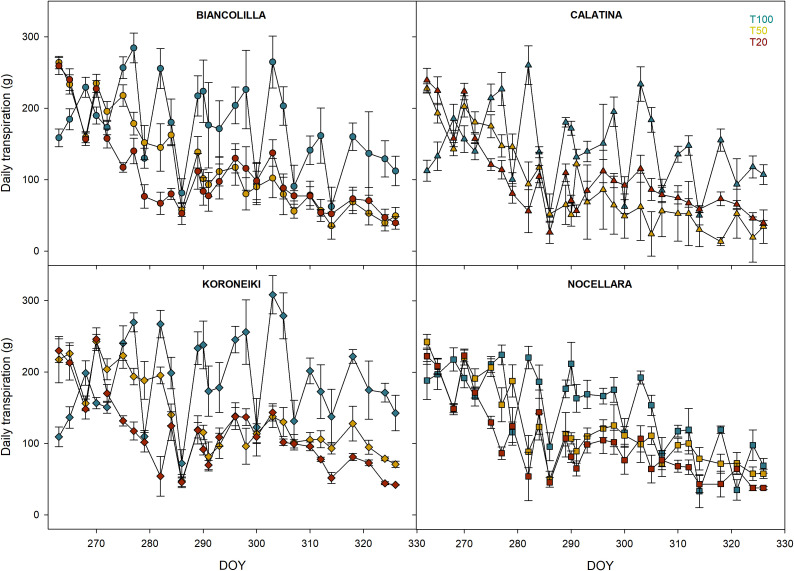
Daily transpiration trend by treatments in Biancolilla, Calatina, Koroneiki, and Nocellara. Error bars indicate standard errors of means. Blue, yellow, and red represent the irrigation treatments T100, T50, and T20, respectively.

Throughout the experimental period, the transpiration rate of each pot was monitored to observe how the different cultivars reacted to varying levels of water stress (**Figure 6**). Overall, daily transpiration closely followed the seasonal trends in atmospheric demand (**Figure 1**). In all the cultivars, the first differences in Ψ_stem_ among treatments were observed at DOY 277 (**Figure 2**); therefore, before that day, the transpiration rate of the treatments (T100, T50, and T20) was not different from each other. Between DOY 280 and 290, elevated temperatures and VPD promoted increased transpiration rates, particularly in well-watered plants (T100) across all cultivars. However, despite temperatures remaining high in the following days, a marked decrease in transpiration was observed at DOY 286. Under the stressed treatments (T50 and T20), plants maintained lower transpiration rates throughout the period, despite the high evapotranspiration demand applied by the environment, likely due to more active stomatal regulation aimed at preserving water. As the season continued, a general decline in temperature and VPD led to a gradual reduction in transpiration across all treatments, particularly on T100. In the final part of the experiment, sharp decreases in transpiration were observed on specific days (**Figure 6**).

**Figure 7:**
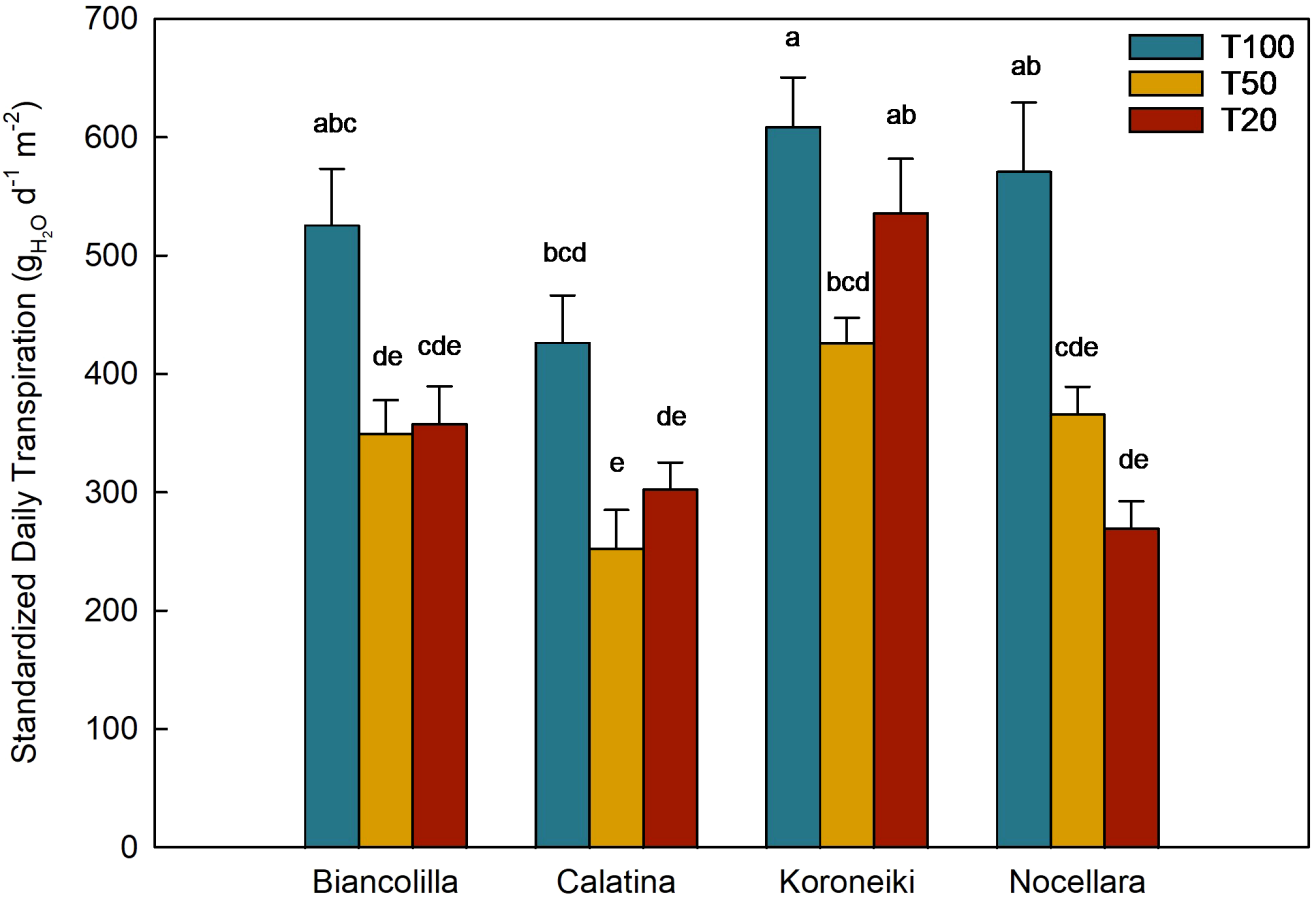
Standardize daily transpiration (STD) in the different cultivars and treatments (mean from DOY 290 to 326). Blue, yellow, and red bars represent the treatments T100, T50, and T20, respectively. Error bars indicate standard errors of mean; n.s., not significantly different; letters mean significant differences for P ≤ 0.05.

Considering the water availability influence on the canopy surface area (**Figure 4**), the standardized daily transpiration (SDT) data from only the last month (from DOY 290 to 326) were analyzed (**Figure 7**) to further investigate cultivar-specific response to water loss.

The SDT was significantly affected by both irrigation treatment and cultivar (**Figure 7**). Under full irrigation (T100), ‘Koroneiki’ exhibited the highest transpiration rate, followed by ‘Nocellara’, ‘Biancolilla’, and ‘Calatina’. As water availability decreased (T50 and T20), a general reduction in SDT was observed across all cultivars. Specifically, even under T20 conditions, ‘Koroneiki’ maintained comparatively high transpiration rates, conversely, ‘Calatina’ displayed a significant decrease in SDT. ‘Nocellara’ exhibited a more gradual reduction in transpiration, while ‘Biancolilla’ showed a sharp decline between T100 and the two stressed treatments.

Since the Ψ_stem_ showed statistically significant differences between treatments on DOY 277, both net photosynthesis (A_n_) and stomatal conductance (g_s_) were, on average, higher in T100 compared to the stressed treatments (T50 and T20; **Table 3**). The ratio C_i_/g_s_ was lower in the well-watered treatment and higher in the stressed ones. No statistically significant differences have been found in C_i_ and WUE_i_, both in cultivars and treatments.

**Table 3:** Mean values (± standard error) of gas exchange parameters.

Parameters		Assimilation (µmol CO_2_ m^-2^ s ^-1^)				Stomatal conductance (mmol H_2_O m^-2^ s^-1^)				Substomatal CO_2_ concentration (µmol mol^-^¹)				WUE_i_ (µmol CO_2_ mmol^-^¹ H_2_O)				C_i_/g_s_			
		Means		S.E.		Means		S.E.		Means		S.E.		Means		S.E.		Means		S.E.	
Cultivar	Biancolilla	12.168	±	0.746	n.s.	93.863	±	4.849	ab	162.913	±	9.844	n.s.	0.128	±	0.006	n.s.	1.961	±	0.176	n.s.
	Calatina	11.219	±	1.009	n.s.	84.609	±	5.956	b	172.741	±	22.259	n.s.	0.107	±	0.014	n.s.	2.737	±	0.877	n.s.
	Koroneiki	13.615	±	0.917	n.s.	102.59	±	5.64	a	139.792	±	10.481	n.s.	0.135	±	0.009	n.s.	1.419	±	0.144	n.s.
	Nocellara	11.63	±	0.844	n.s.	88.002	±	6.186	ab	152.207	±	13.06	n.s.	0.137	±	0.011	n.s.	1.675	±	0.157	n.s.
Treatment	T100	16.116	±	0.615	a	118.302	±	3.89	a	136.746	±	6.198	n.s.	0.138	±	0.004	n.s.	1.193	±	0.059	b
	T50	10.081	±	0.773	b	79.196	±	4.96	b	172.292	±	16.637	n.s.	0.116	±	0.010	n.s.	3.501	±	0.918	a
	T20	10.074	±	0.553	b	78.255	±	3.714	b	163.188	±	12.93	n.s.	0.126	±	0.011	n.s.	2.141	±	0.159	ab
Cultivar*Treatment		n.s.				n.s.				n.s.				n.s.				n.s.			

Different letters indicate statistically significant differences between means according to Tukey’s HSD test (P ≤ 0.05); n.s, not significantly different.

**Figure 8:**
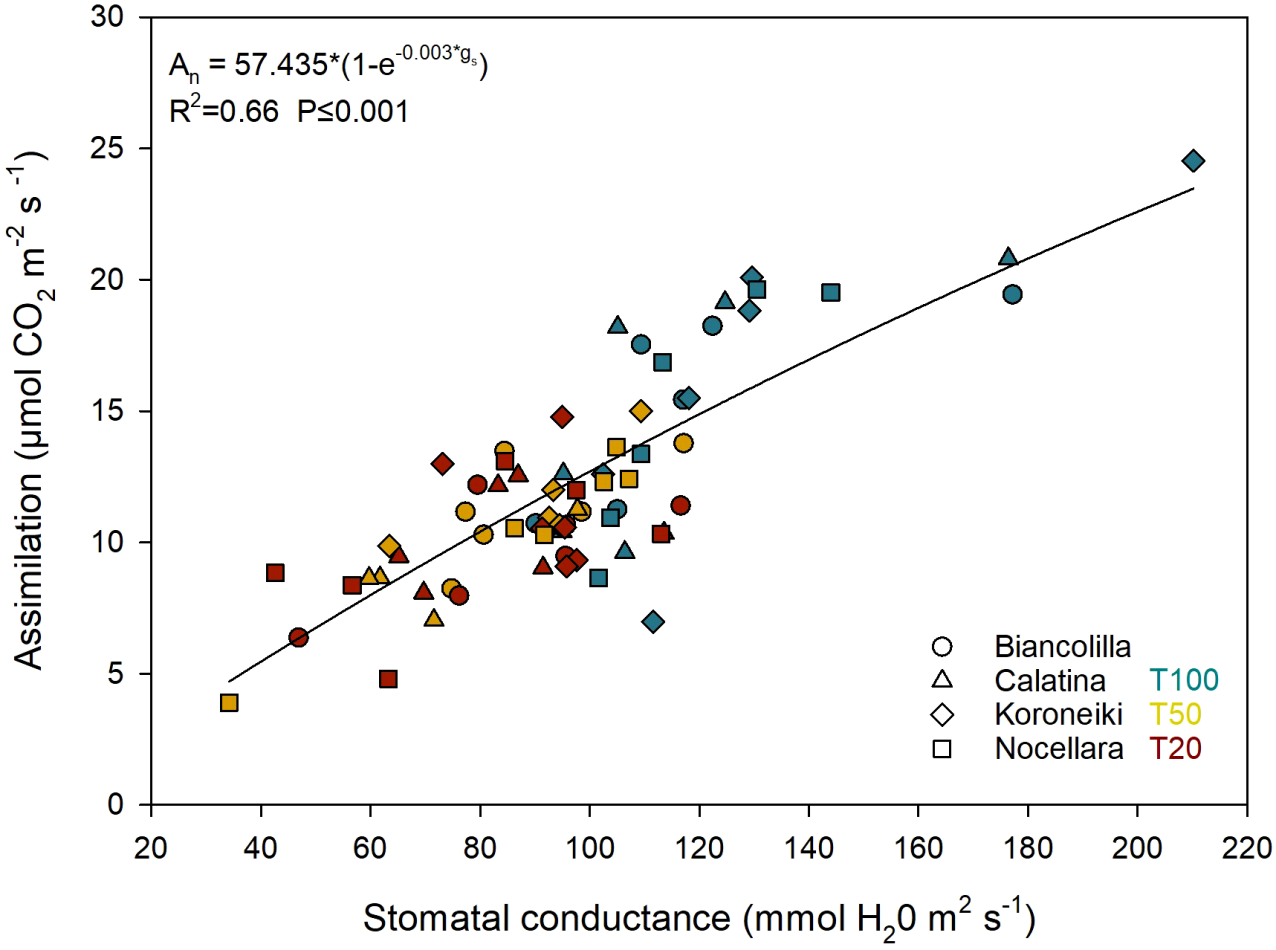
Relationship between stomatal conductance (g_s_) and assimilation (A_n_). Different shapes of the dots represent the cultivars. Blue, yellow, and red represent the irrigation treatments T100, T50, and T20, respectively.

Among the cultivars, only g_s_ reported statistically significant differences (P ≤ 0.001; **Table 3**). On average, ‘Calatina’ regulated stomatal aperture more restrictively than ‘Koroneiki’. In contrast, ‘Biancolilla’ and ‘Nocellara’ exhibited intermediate values. The overall relationship between A_n_ and g_s_ was also plotted (**Figure 8**), revealing a significant nonlinear relationship (R² = 0.66).

**Figure 9:**
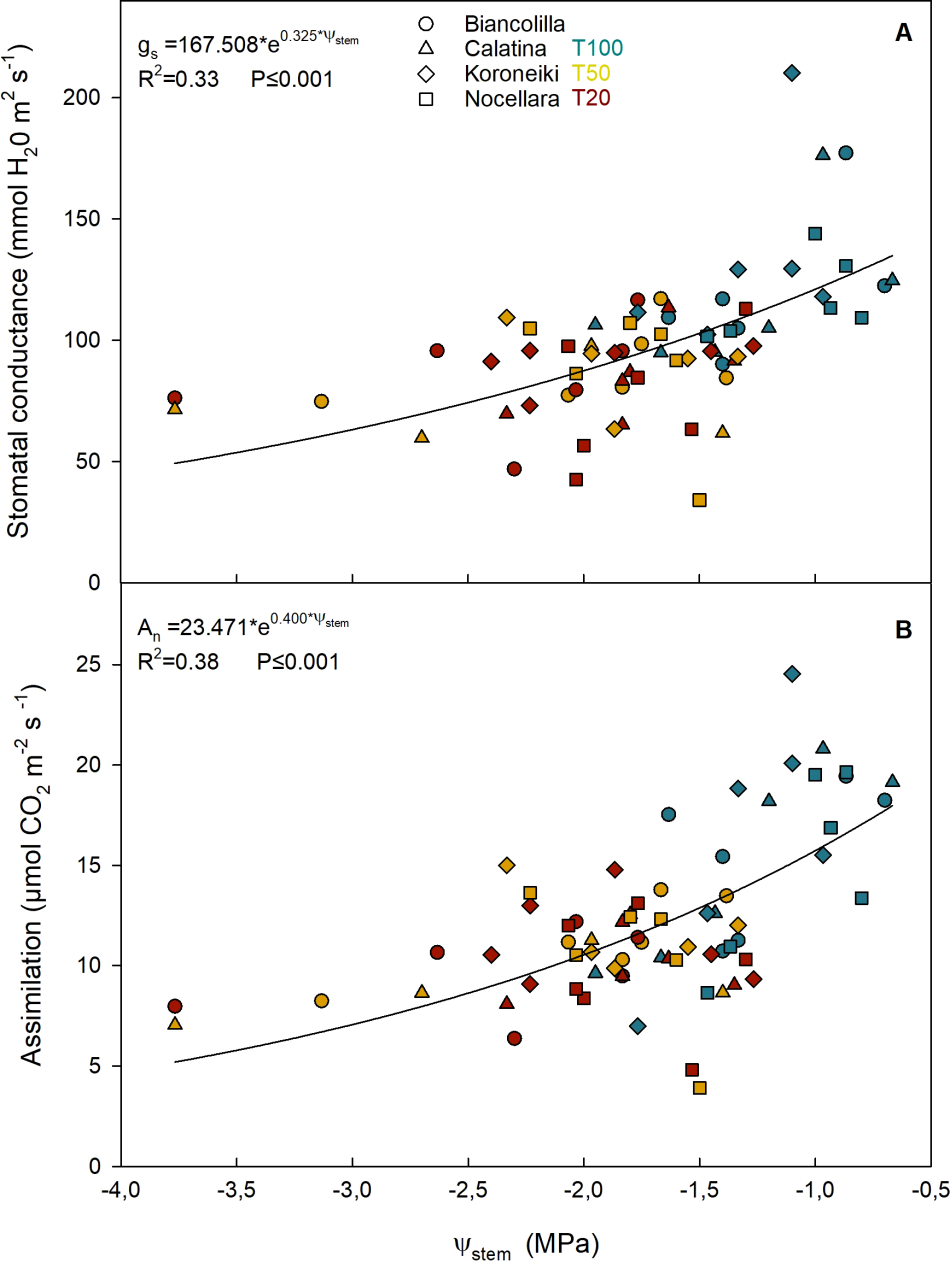
Relationship between midday stem water potential (Ψ_stem_) and stomatal conductance [gs; **(A)**], and assimilation [An; **(B)**]. Different shapes of the dots represent the cultivars. Blue, yellow, and red represent the irrigation treatments T100, T50, and T20, respectively.

**Figure 10:**
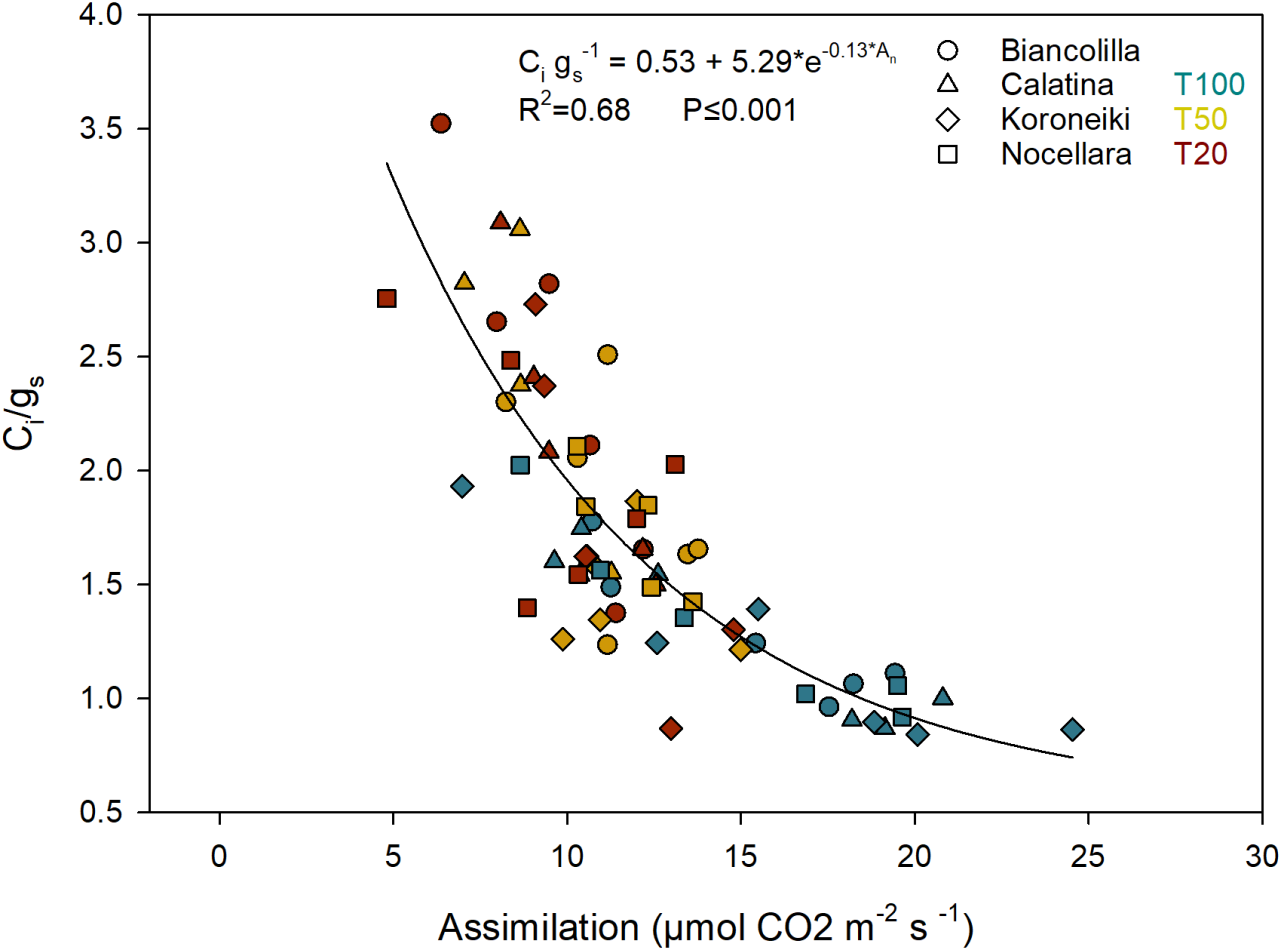
Relationship between the assimilation (A_n_) and the indicator of mesophyll efficiency (C_i_/g_s_). Different shapes of the dots represent the cultivars. Blue, yellow, and red represent the irrigation treatment T100, T50, and T20, respectively.

Both g_s_ and A_n_ exhibited an exponential response to Ψ_stem_ (**Figure 9**). The g_s_ response curve highlights a progressive reduction in stomatal sensitivity under increasing water stress, with values stabilizing around 50–70 mmol m^-^² s^-^¹ even under severe conditions (**Figure 9A**). Similarly, A_n_ declined with decreasing Ψ_stem_. The ratio C_i_/g_s_ ​ was plotted against the An (**Figure 10**) to evaluate how the mesophyll efficiency supports photosynthetic performance. An inverse exponential relationship was observed (R² = 0.68), indicating that as assimilation increases, the intercellular CO_2_ concentration (C_i_) decreases relative to a given stomatal conductance (g_s_). In particular, the T100 treatments exhibited lower C_i_/g_s_ values (less than 2). Conversely, higher C_i_/g_s_ values were recorded in the stressed plants.

### 3.5 Effect of water stress on hydraulic conductance

**Figure 11:**
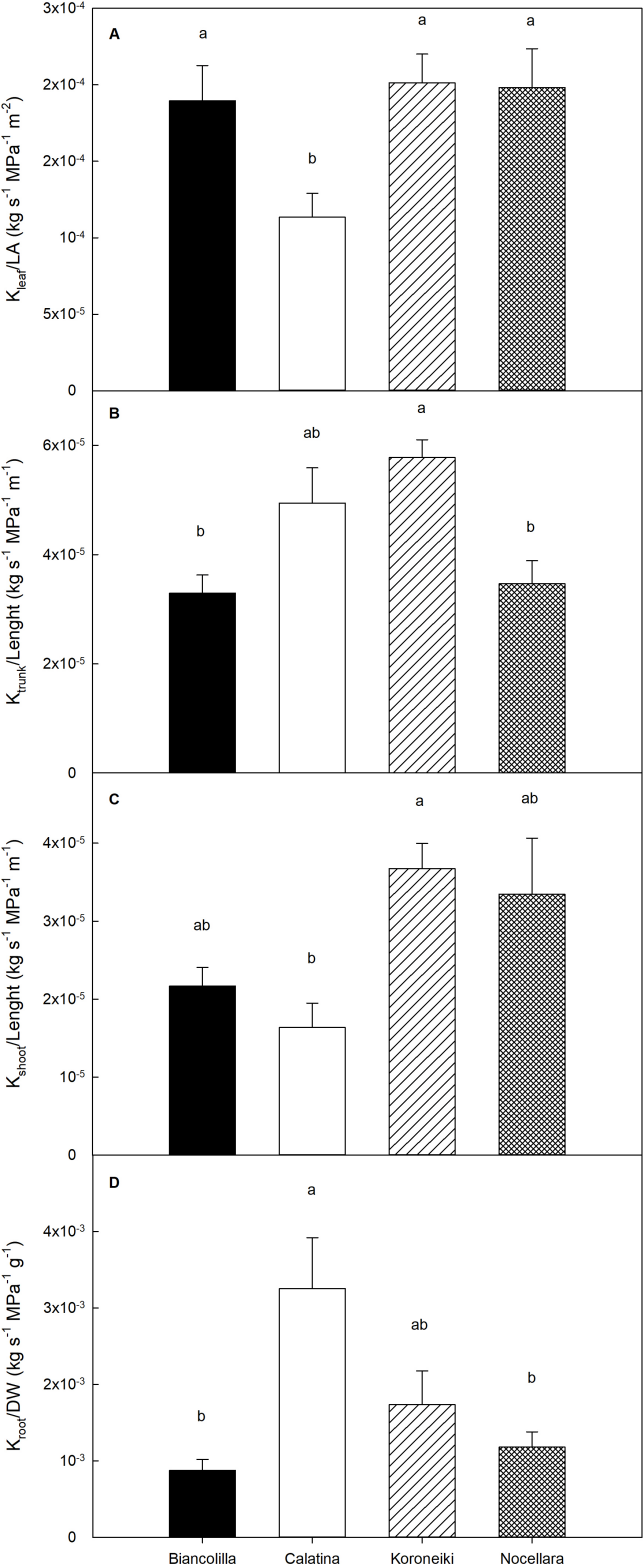
Hydraulic conductance of leaf **(A)**, trunk **(B)**, shoot **(C)**, and root **(D)** of the trees, normalized by the corresponding biometric parameter. Error bars indicate standard errors of means. n.s., not significantly different; letters, significantly different for P ≤ 0.05.

Statistically significant differences were found among cultivars for k_leaf_, k_shoot_, k_trunk_, and k_root_ (**Figure 11**). Specifically, ‘Biancolilla’, ‘Koroneiki’, and ‘Nocellara’ exhibited markedly higher leaf hydraulic conductance (k_leaf_) compared to ‘Calatina’ (**Figure 11A**). Regarding k_shoot_ the cultivar Koroneiki recorded the highest hydraulic conductance, followed by ‘Nocellara’ and ‘Biancolilla’, while Calatina exhibited the lowest (**Figures 11C**). For the hydraulic conductance of the trunk (k_trunk_), the highest value was again observed in ‘Koroneiki’, but in this case ‘Biancolilla’ and ‘Nocellara’ recorded the lowest conductance (**Figure 11B**). Finally, for root hydraulic conductance (k_root_), ‘Calatina’ showed the highest value, followed by ‘Koroneiki’, whereas ‘Biancolilla’ and ‘Nocellara’ showed consistently lower k_root_ values (**Figure 11D**). Overall, these results highlight cultivar-specific differences in hydraulic conductance across organs.

## 4 Discussions

### 4.1 Effect of water stress on the biomass partitioning and morphological traits

The results revealed clear genotype-dependent responses in biomass allocation and vegetative growth traits under water stress. These findings are discussed below in relation to cultivar vigor, growth habits, and drought adaptation strategies.

Water stress had a clear and significant effect on the growth of the canopy, particularly on the number of leaves of plants under severe water deficit, but also on the single leaf area (**Table 2**). The reduction in available water significantly affected the canopy surface area of ‘Koroneiki’ (**Figure 4**), suggesting that its canopy development is particularly sensitive to drought. Given that leaves are the most plastic organs in response to drought conditions (El Bey et al., 2024; Ennajeh et al., 2010), these differences are important for cultivar characterization (Caruso et al., 2007).

The biomass allocation was influenced by the treatment and cultivar (**Figure 3**). The observed stability of root biomass across treatments (**Figure 3D**) suggests that the olive root may be less sensitive to prolonged water deficit compared to aboveground organs, possibly as a drought adaptation strategy. Previous studies (Polverigiani et al., 2011) confirm that olive roots, particularly pioneer roots, exhibit strong plasticity and maintain their function under variable soil moisture conditions. Furthermore, Bacelar et al. (2007) showed that preserving root growth under stress is associated with improved hydraulic efficiency and water use, thereby reinforcing the idea that root maintenance is key to drought resilience. The patterns of the relative biomass allocation reflected the different growth habits and architectural traits of the cultivars (**Table 1**). ‘Calatina’ showed a dwarfing habit and allocates more biomass to canopy growth rather than trunk elongation. This characteristic aligns with its potential suitability for high-density orchards, where maximizing lateral growth for light interception is favored (Marino et al., 2019; Massenti et al., 2022). Conversely, the investment focused on the trunk biomass observed in ‘Biancolilla’ and ‘Nocellara’ may also be linked to their recognized high vigor (Sofo et al., 2009; Massenti et al., 2024), while ‘Koroneiki’ appeared to adopt a more belowground-focused strategy, possibly enhancing drought resilience.

In our study, as water stress increased, plants allocated more biomass to the roots and reduced the biomass of the leaves. This pattern supports the findings of Chaves et al. (2003), who reported that, under drought conditions, plants tend to minimize water loss and enhance water uptake. In particular, one strategy to minimize water loss is to reduce canopy growth, while water uptake can be optimized by adjusting the allocation pattern, specifically by increasing investment in the roots (Jackson et al., 2000).

The differences in trunk cross-section area (TCSA) confirmed the vigor levels of the cultivars reported in the literature (**Figure 5**). ‘Calatina’, the most promising local accession for mechanization (Marino et al., 2019; Massenti et al., 2022), confirmed its low vigor, showing minimal TCSA change between the start (DOY 261) and end (DOY 326) of the experiment. In contrast, ‘Biancolilla’ confirmed its status as the most vigorous cultivar (Sofo et al. 2009) among the autochthonous varieties, followed by ‘Nocellara’. Interestingly, ‘Koroneiki’, which had initial TCSA values similar to ‘Calatina’ and ‘Nocellara’, exhibited the largest increase by the end of the trial, surpassing even ‘Biancolilla’. Similar results were reported by Pérez-Rodríguez et al. (2024) and Cuevas et al. (2024), defining ‘Koroneiki’ as a vigorous cultivar.

The results of the shoot growth (**Table 2**) were consistent with previous studies showing how plant architecture can be affected by water shortages, leading to reductions in both size and dry matter (Xiloyannis et al., 1999; Dichio et al., 2002; Bacelar et al., 2007; Di Vaio et al., 2012; 2013; Rosati et al., 2024), especially in the shoot elongation, which was defined as the most drought-sensitive growth parameter in olive trees by Sofo et al. (2008) and Ben-Gal et al. (2010).

Finally, the canopy-to-root mass ratio decreased as water deficit increased (**Table 2**), consistent with the findings of Dichio et al. (2002), who observed that in olives, lower water availability leads to a greater reduction in the growth of aboveground organs compared to underground organs. This ratio is a crucial indicator of the balance between water absorption and the transpiring surface area. Therefore, under water deficit conditions, reducing the canopy-to-root mass ratio improves water availability per unit of leaf area, helping plants maintain photosynthesis during prolonged droughts (Dichio et al., 2002). An imbalance between these components can compromise water relations, reduce stress tolerance, and ultimately affect productivity and survival (Coleman and Isebrands, 1994; Garcia-Tejera et al., 2018).

### 4.2 Effect of water stress on gas exchanges and transpiration rate

The decrease in transpiration observed at DOY 286 in T100 (**Figure 6**) suggests the involvement of additional limiting factors, such as the drop in VPD due to increased relative humidity, or a physiological adjustment by the plants, including partial stomatal closure to avoid excessive dehydration under prolonged thermal stress (Haworth et al., 2018). These dynamics show the strong influence of environmental conditions on transpiration, and the skill of plants to adjust their water use in response not only to soil water availability but also to prolonged exposure to high atmospheric demand. In T50 and T20, low and continued transpiration rates were probably due to more active stomatal regulation aimed at preserving water.

To further investigate the cultivar-specific response in loss of water by transpiration, the data from the last month (from DOY 290 to 326) were analyzed (**Figure 7**). The findings showed an elevated transpiration rate in all cultivars when fully irrigated (T100), indicating a good capacity for water uptake and transpiration under optimal conditions, and a common reduction when water stress increased. However, among the cultivars, different approaches to water stress were observed: Koroneiki maintained a high transpiration rate also in T20, showing less control in water loss when the water availability decreases; on the contrary, Biancolilla, Calatina, and Nocellara significantly reduced the transpiration suggesting a more conservative approach to drought conditions. This observation may indicate slight variations in stomatal regulation or hydraulic conductance. The ability of these cultivars to regulate their transpiration under stress, without completely shutting down gas exchange, may represent a compromise strategy between drought avoidance and maintenance of physiological activity (Carella et al., 2024).

Considering that g_s_ highly controls A_n_ in olive (Angelopoulos et al., 1996; Tognetti et al., 2007), the two parameters were related in **Figure 8**. Moriana et al. (2002) observed a linear relationship between stomatal conductance and photosynthesis. However, a nonlinear relationship was used (**Figure 8**), as suggested by Ahumada-Orellana et al. (2019), who observed that when g_s_ values exceeded 200 mmol m^-2^ s^-1^, the relationship tended to become nonlinear.

To understand the physiological response of olive trees to water availability, the relationships between Ψ_stem_ and two key gas exchange parameters-net CO_2_ assimilation rate (A_n_) and stomatal conductance (g_s_) were analyzed (**Figure 9**). Previous studies have reported a significant correlation between g_s_ and Ψ_stem_ in olive trees, highlighting the sensitivity of stomatal behavior to plant water status (Fernández et al., 2006; Tognetti et al., 2007; Ben-Gal et al., 2009, 2010; Marino et al., 2016). The decline of g_s_ and A_n_ with -2.5 MPa Ψ_stem_ confirms this threshold as a critical limit for gas exchange processes in olive (Marino et al., 2018).

Both g_s_ and A_n_ exhibited an exponential response to Ψ_stem_. When Ψ_stem_ dropped below -2.5 MPa, a marked decline in both parameters was observed. The g_s_ response curve highlights a progressive reduction in stomatal sensitivity under increasing water stress, with values stabilizing around 50–70 mmol m^-^² s^-^¹ even under severe conditions (**Figure 9A**). Similarly, A_n_ declined with decreasing Ψ_stem_, although the relationship remained moderate (R² = 0.38), indicating that photosynthetic activity is partially maintained even under stress, possibly due to metabolic or osmotic adjustments (Gucci and Caruso, 2010; **Figure 9B**). These trends are consistent with previous findings in olive trees (Angelopoulos et al., 1996; Ennajeh et al., 2008; Marino et al., 2018; Ahumada-Orellana et al., 2019; Carella et al., 2024), where gas exchange is gradually downregulated, rather than suddenly, under drought, supporting the hypothesis of a compromise strategy that allows the plant to preserve core physiological activity while limiting water loss.

High photosynthetic activity is positively correlated with high stomatal conductance and also with lower CO_2_ concentrations in the stomatal cavity. According to Sheshshayee et al. (1996), the C_i_/g_s_ ratio could serve as a rapid indicator of mesophyll efficiency and a measure of mesophyll control over photosynthesis (Chandra et al., 2008; Bindumadhava et al., 2007). Thus, the diffusion of CO_2_ into the sub-stomatal cavity reflects the intrinsic capacity of the mesophyll to support photosynthesis.

In **Figure 10**, C_i_/g_s_ ratio low values (less than 2) in the T100 treatment suggest that, for a given stomatal opening (g_s_), the plant is keeping more CO_2_ inside the stomatal cavity, potentially a sign of high photosynthetic efficiency or stomatal limitation. The higher C_i_/g_s_ ratio, observed in stressed plants, could indicate that CO_2_ is not being efficiently utilized for photosynthesis due to biochemical limitations in the photosynthetic apparatus. Similar results were observed by Ramanjulu et al. (1998) in mulberry. As suggested by Sheshshayee et al. (1996), this parameter may be useful for the early detection of high stress levels caused by drought.

### 4.3 Effect of water stress on hydraulic conductance

Hydraulic conductance is closely linked to photosynthesis and growth (Tyree, 2003; Nardini et al., 2006; Rewald et al., 2011). The limited impact of irrigation treatments on hydraulic traits may be attributed to the high drought tolerance of the species (Baccari et al., 2020). This suggests that the stress levels reached, in terms of Ψ_stem_ (**Figure 2**), probably were not sufficient to induce significant differences among the treatments. Research on the hydraulic architecture of olives is limited (Nardini et al., 2006; Raimondo et al., 2009), and comparisons among different cultivars are lacking. Integrating hydraulic conductance data with biomass allocation, gas exchange, and daily transpiration highlights contrasting strategies among cultivars.

It appears that in ‘Koroneiki’, water is transported efficiently by all epigeal organs (high k_leaf_, ks_hoot_ and k_trunk_), while it is transported with slightly more difficulty by the root system (low k_root_) (**Figure 11**). k_leaf_ is considered the hydraulic capacity of the stem to supply leaves with water (Sterck et al., 2008; Dichio et al., 2013) and was consistent with the range reported by Raimondo et al. (2009). Thus, this behavior reflects an efficient water transport system that supports high transpiration rates, even under conditions of limited water availability (Scalisi et al., 2019; Hernandez-Santana et al., 2016; **Figure 7**). The ability to support high water fluxes also promotes continuity of photosynthetic activity and carbon allocation for growth processes. Throughout the trial, ‘Koroneki’ maintained an average photosynthesis of 13.615 ± 0.917 µmol m^-2^ s^-1^, the highest g_s_ value (**Table 3**) and also exhibited an increase in trunk diameter (**Figure 5**), suggesting greater cambial activity associated with increased hydraulic demand.

In contrast to ‘Koroneiki’, ‘Calatina’ reported higher hydraulic conductance in the roots and lower hydraulic conductance in the epigeal zone (particularly in leaves and shoots). This habit suggests a strongly conservative water strategy: while water uptake remains efficient, its transport to the upper parts of the plant is tightly controlled. Evidence of its active stomatal regulation is supported by the low average g_s_ value (84.609 ± 5.956 µmol m^^-^²^ s^^-^¹^), the lowest recorded among the cultivars, as well as by the SDT shown in **Figure 7**, which highlights control over transpiration rates. These results are in accordance with Nardini and Salleo (2000) and Vilagrosa et al. (2012), who observed that shoot hydraulic conductance limited the maximum stomatal conductance, confirming that in olive, g_s_ is strongly related to k_leaf_ (Hernandez-Santana et al., 2016). This hydraulic configuration may help the plant minimize water loss under drought conditions, but it could also limit photosynthesis and growth (Bacelar et al., 2007; Solari et al., 2006), suggesting that this strategy could be a contributing factor to its low vigor and slower growth rate compared to other cultivars (**Figure 5**). Nevertheless, this finding contrasts with the conclusions of Nardini et al. (2006), who argue that low root hydraulic conductance is the primary factor leading to reduced vegetative growth in grafted olive trees.

However, several studies have found that the hydraulic limitation of plant growth is related to a reduction in leaf-specific hydraulic conductance (Schäfer et al., 2000; Mencuccini, 2003; Bacelar et al., 2007; Solari et al., 2006), as well as has been ‘observed in ‘Calatina’.

In ‘Biancolilla’ and ‘Nocellara’, the hydraulic architecture indicated a more controlled water transport system that minimizes excessive water loss, particularly under stress conditions. In ‘Nocellara’, the transpiration rates (**Figure 7**) showed a gradual decrease, suggesting a moderately conservative response to drought. Despite this adjustment, the cultivar maintained a balanced growth pattern (**Figure 3**) and stable biomass partitioning, which may reflect an adaptive compromise between productivity and water conservation. In ‘Biancolilla’, the hydraulic architecture suggested that leaf-level water transport remains efficient under optimal conditions, but the overall water flow through the plant is actively downregulated as stress increases. The significant reduction in transpiration observed from T100 to T50, followed by a stable phase at T20 (**Figure 7**), supports the idea of strong stomatal regulation. This phenomenon indicates that the plant rapidly reduces water use in response to a decrease in soil moisture and thus stress (Testi et al., 2006).

## 5 Conclusion

In the current Mediterranean context, one of the most pressing challenges is the increase in temperatures, often accompanied by a high vapor pressure deficit (VPD), which leads to elevated evapotranspirative demand on plants. Identifying olive cultivars that can adapt to such conditions without significant yield or quality losses is essential for sustaining production in the face of climate change. This study highlights the presence of marked differences among cultivars in their physiological and morphological responses to progressive water stress. Daily transpiration patterns closely reflected changes in atmospheric demand, particularly during the initial stages of the experiment when air temperatures and VPD reached very high levels. However, under prolonged stress, cultivar-specific strategies became evident. ‘Koroneiki’ showed high conductance in aerial parts, supporting sustained photosynthesis and growth even under limited water availability. In contrast, ‘Calatina’ displayed a more conservative pattern, with high root conductance but restricted water transport to shoots and leaves, a strategy consistent with water-saving behavior and reduced photosynthetic performance. The intermediate behavior observed in ‘Biancolilla’ and ‘Nocellara’ reflects a regulated hydraulic architecture that balances productivity and drought avoidance.

The distribution of biomass and plant growth were influenced by both cultivar and irrigation treatments. As water stress increased, a progressive reduction in biomass was observed in all organs, particularly in shoots in ‘Biancolilla’ and ‘Koroneiki’, which reported a reduction in growth of 60% in comparison to the well-irrigated plants. The cultivars also differed in their biomass allocation strategy: for instance, ‘Calatina’ invested more in shoots and less in the trunk, confirming its dwarfing habit, which is helpful for high-density plantings, while ‘Biancolilla’ and ‘Nocellara’ invested more in the trunk, proving themselves as vigorous cultivars, as well as ‘Koroneiki’, which reported the highest TCSA. The root system exhibited less sensitivity to water shortage, suggesting a possible adaptive mechanism that maintains water uptake. Reduction in irrigation also affected the canopy-to-root mass ratio. The balance between the canopy and roots is crucial for water-use efficiency and stress resilience.

These findings can support the selection of genotypes better suited to future climatic scenarios, contributing to the resilience of Mediterranean olive production systems. Cultivars with adaptive morphologies, efficient stomatal control, and hydraulic architecture may offer advantages in water-limited environments by optimizing water use and minimizing stress-related damage. A deeper understanding of the hydraulic characteristics of Italian olive cultivars may help guide their use in agronomic systems designed for local water availability and climate conditions. While this experiment provided valuable insights under controlled conditions, future research should evaluate long-term effects on yield and oil quality in field environments. Understanding genetic diversity in drought response is essential for the future of Mediterranean agriculture.

## Data Availability

The raw data supporting the conclusions of this article will be made available by the authors, without undue reservation.
